# Complications in Post-Liver Transplant Patients

**DOI:** 10.3390/jcm12196173

**Published:** 2023-09-24

**Authors:** Carlotta Agostini, Simone Buccianti, Matteo Risaliti, Laura Fortuna, Luca Tirloni, Rosaria Tucci, Ilenia Bartolini, Gian Luca Grazi

**Affiliations:** Department of Experimental and Clinical Medicine, AOU Careggi, 50134 Florence, Italy; carlotta.agostini@unifi.it (C.A.); simone.buccianti@unifi.it (S.B.); matteorisaliti@gmail.com (M.R.); laura.fortuna@unifi.it (L.F.); luca.tirloni@unifi.it (L.T.); rosaria_tucci@libero.it (R.T.); gianluca.grazi@unifi.it (G.L.G.)

**Keywords:** liver transplantation, postoperative morbidity, surgical complications, medical complications

## Abstract

Liver transplantation (LT) is the treatment of choice for liver failure and selected cases of malignancies. Transplantation activity has increased over the years, and indications for LT have been widened, leading to organ shortage. To face this condition, a high selection of recipients with prioritizing systems and an enlargement of the donor pool were necessary. Several authors published their case series reporting the results obtained with the use of marginal donors, which seem to have progressively improved over the years. The introduction of in situ and ex situ machine perfusion, although still strongly debated, and better knowledge and treatment of the complications may have a role in achieving better results. With longer survival rates, a significant number of patients will suffer from long-term complications. An extensive review of the literature concerning short- and long-term outcomes is reported trying to highlight the most recent findings. The heterogeneity of the behaviors within the different centers is evident, leading to a difficult comparison of the results and making explicit the need to obtain more consent from experts.

## 1. Introduction

Liver transplantation (LT) is the therapy of choice for liver failure [1]. Approximately 140,000 LTs are performed per year, covering approximately 10% of the demand. Since the first orthotopic LT was performed in the 1960s [2], indications have been expanded but the most common are chronic and acute liver failure. Cirrhosis accounts for almost 80% of the causes of LT. Hepatitis C virus (HCV) infection is the actual leading cause of cirrhosis and a risk factor for the development of hepatocellular carcinoma (HCC), which is a potential indication for LT [3]. The introduction of viral eradicating programs will probably reduce the frequency of this infection shortly. Other indications to LT include mass syndrome in autosomal dominant polycystic kidney disease (ADPKD) and some other inherited diseases. Primary hyperoxaluria type 1 is an autosomal recessive disease induced by the impaired function of the alanine-glyoxylate aminotransferase, leading to end-stage renal disease and oxalosis. In the early stages of the disease, LT could be an option to preserve renal function fixing metabolic defects; otherwise, a combined liver–kidney transplantation may be required [4,5]. Familial amyloid polyneuropathy is an autosomal dominant inherited disorder provoked by the mutation of transthyretin (TTR). Mutated TTR produced by the liver is a precursor of amyloid and tissue accumulation of amyloid causes peripheral and autonomic polyneuropathy. LT in the early stage of the disease is reported to significantly lengthen survival [6] In this setting, domino transplantation could be a valuable option [7]. Finally, some kinds of liver malignancies, including unresectable Klatskin tumors or selected cases of metastases, may be evaluated for LT, mostly within clinical trials [8,9,10].

Since organ demand outperforms organ supply, several scores have been proposed to establish the priority for LT and to choose the best donor/recipient match. The Model for End-Stage Liver Disease (MELD) score quantifies the severity of chronic liver disease. Several slightly modified versions have been proposed over the years [11,12].

To face the increased demand for organs, the medical community tried to extend the donor criteria and the donor pool with the so-called marginal or extended criteria donor (ECD) that also includes Donation after Circulatory Death (DCD). The latter category includes two different kinds of donors: controlled DCD (cDCD), donation after the programmed withdrawal of life-sustaining therapies, and uncontrolled DCD (uDCD), donation after unsuccessful advanced cardiopulmonary reanimation. Although DCD raises several ethical and legal issues in different countries, DCD represents an effective way to increase the donor pool. An expansion in DCD was observed over the last decade, even if the liver discard rate is higher for DCD when compared to DBD [13].

Outcomes following donation after brain death (DBD) and DCD are reported to be quite similar except for a higher rate of short-term medical complications after DCD due to the longer ischemia time [14,15]. Similar results may be achieved using ECD in specialized centers, although there is complete disagreement on the definition of ECD [16]. In these cases, an accurate selection of donor/recipient match is of utmost importance to achieve acceptable results. The UK-DCD score represents a potential tool to avoid high-risk donor/recipient matches. It includes donor age and BMI, recipient MELD score and age, history of a previous LT, donor functional warm ischemia, and cold ischemia. Patients with a score higher than 10 are classified as a “futile” group [17]. Other scores and allocation methods have been proposed over the years with their strengths and disadvantages [18].

In situ organ preservation maneuvers (Extracorporeal Membrane Oxygenation support with normothermic abdominal organ perfusion—NRP) reduce ischemic damage, provide oxygen and nutrients to the liver, and eliminate toxic metabolites before graft storage, allowing similar results to DBD [19]. Advanced ex situ organ preservation procedures (including normothermic machine perfusion—NMP—or hypothermic oxygenated machine perfusion—HOPE) are still a matter of debate. Still, they seem to allow donor pool expansion (including donors older than 80 years), to improve organ viability and LT outcomes over static cold storage (SCS) [20,21,22,23,24]. Furthermore, ex situ organ perfusion systems offer the possibility to test organ function, more time to choose the best donor/recipient match, and to perform LT [20,21,22]. Potential bile biomarkers able to assess graft viability, biliary tree function, and predict possible biliary complications are under evaluation [25].

The use of split liver (both from living or dead donors) is another possibility to increase graft availability [3]. Although survival rates are higher after living donor LT (LDLT) [26], potential donor complications with a reported morbidity and mortality rate of 30% and 0.8%, respectively, should be carefully considered [3].

The actual morbidity rate is relatively low, but complications, mostly occurring during the first month, are still the primary cause of postoperative death. The great majority of the patients are strictly monitored in Intensive Care Units after LT, where metabolic and electrolyte status, together with graft synthetic function, are closely reassessed and corrected [27]. Improvements in perioperative management, together with a broader knowledge of postoperative morbidities and advances in imaging techniques, led to an early diagnosis and treatment of the complications with a better prognosis for the patients [28]. Globally, the 1- and 10-year survival rates have reached 96% and 71%, respectively [1]. As a consequence of the expansion of the indications and better outcomes, the medical community has to face the management of long-term complications after LT [12].

The incidence, risk factors, and management of postoperative and long-term complications will be discussed below, trying to highlight the most recent findings. However, most of the published data reported single-institution series. This aspect, together with a wide range of behaviors in every step of the transplantation process, leads to a problematic comparison and interpretation of the data.

## 2. Methods

“Liver transplantation” combined with the keywords “postoperative morbidity”, “surgical complications”, or “medical complications” was used to perform extended research on PubMed, Cochrane Library, and Scopus. The bibliography of each article was also screened. Duplicated manuscripts were excluded. Only manuscripts written in English were evaluated.

## 3. Medical Complications

### 3.1. Primary Graft Dysfunction

Early allograft dysfunction (EAD) is a dangerous complication with an incidence of 5.2–40% [29,30]. Primary nonfunction (PNF) is the most serious form of this complication.

Several definitions of EAD exist [31]. The introduction of the Model of Early Allograft Function score also allowed the quantification of the dysfunction using a scale ranging from 0 to 10. Parameters evaluated are the maximum level of alanine aminotransferase, INR, and bilirubin within the first 3 days after surgery [32]. This score also has a prognostic value for patient survival within the first year after LT [33]. Other predictive scores have been proposed over the years, including the Liver Graft Assessment Following Transplantation (L-GrAFT) risk score, which evaluates the 3-month risk of graft failure [30]. Both cold and warm ischemia time and donor parameters (including age, body mass index—BMI, steatosis, and cause of death) are well-known risk factors for graft function impairment together with other technical aspects [29].

Donor age cut-off is not established, and it has progressively increased over the years with similar outcomes [34]. Schlegel et al. conducted a comparative analysis of DCD younger or older than 60 years. They found comparable graft function, vascular and biliary complication rates between the two groups. At the same time, donor BMI strongly affected the survival rate of the graft and the patients, independently from donor age [35]. However, studies on age impact may suffer from selection bias.

An elevated level of serum sodium in the donor seems to be associated with higher EAD rates [34], although contrasting results may be found in the literature [27].

Recently, donor extraction time (from aortic cross-clamp to liver extraction) has been reported to be another independent prognostic factor related to ischemia time [31]. EAD and PFN have up to 3.6-fold higher incidence after DCD than DBD, but survival rates are comparable with proper perioperative management [14,19,36]. Marcon et al. reported higher but not significant rates of PNF, hepatic artery thrombosis, and ischemic cholangiopathy in recipients of previously discarded livers. This paper underlined the disagreement between specialists, the importance of expertise (including in the procurement phase), and a correct donor/recipient match [16]. Definite evaluations at a molecular level are still missing, but they could provide interesting insights for prevention and treatment [37].

The use of ex situ machine perfusions may be a vital tool to enlarge the graft pool and reduce morbidity after LT. Mergental et al. conducted a study evaluating the results of LT of initially discarded high-risk livers recovered with NMP. All five livers were deemed recovered (based on clearance of lactate in the perfusate 2 h after NMP start), and the transplant success rate in the five patients enrolled was 100%. None of them developed EAD or biliary complications after a follow-up of 24 months [20]. Assessment of lactate clearance, maintenance of pH, production of bile, vascular flow patterns, and evaluation of liver appearance are the most critical aspects of the judgment on organ viability [38]. Other authors proposed the possibility of manipulating the liver content of lipids via NMP, obtaining a reduction of 40% of the steatosis after 6 h of NMP in a small series of 10 patients [39]. Schlegel et al. performed a case-matched comparison between 50 DBD, 50 HOPE-treated DCD, and 50 untreated DCD. They found comparable short- and long-term outcomes between the HOPE-treated DCD and DBD group, while untreated DCD had significantly worse results [40].

Primary nonfunction is caused by hepatic necrosis as the consequence of a severe preservation injury. The reported incidence rate is approximately 5% [27]. Symptoms of PNF include hepatic coma, hemodynamic instability, persistent hypothermia, and renal impairment, causing longer length of hospital stay and higher mortality rates [34]. Laboratory signs include elevated transaminase levels, high bilirubin levels, severe and refractory coagulopathy, hypoglycemia, and lactic acidosis. The level of serum factor V on postoperative day (POD) 1 has been proposed as a precocious biomarker for EAD [41]. Evaluation of the indocyanine green plasma disappearance rate (ICG-PDR) on POD 1 resulted in being a significant and straightforward predictor of EAD development (cut-off value of 16%/min, sensitivity and specificity of 83% and 56%, respectively) and survival at 3 and 12 months and 5 years after LT [42]. Diaz-Nieto et al. proposed a simple score to predict EAD using the peak of AST on POD1, AST reduction within POD 3, and ALT increase or reduction from POD 1 to POD 3. However, this score needs further investigation [43]. An ultrasound evaluation should exclude vascular complications. Plasma exchange may improve patient outcomes, but an urgent re-transplant is required [27].

The “small-for-size syndrome” is represented by liver failure after the transplantation of a split liver. The incidence ranges from 0 to 11% [44]. The ratio between graft and recipient weight is a predictor of this syndrome with a cut-off ranging from 0.6 and 0.8 [45]. The recent concept of the “small-for-flow syndrome” considers the crucial pathogenetic role of portal hyperflow in relation to the volume of the liver [46]. Consequently, prevention can be made with intraoperative flow modulation. Clinical manifestations include cholestasis, alteration of the coagulation, and signs of portal hypertension [45]. Treatments aim to reduce portal hypertension, mostly with the sacrifice of the spleen. However, they are often ineffective, and the mortality rate is still high (up to 50%). The use of potentially small grafts should be definitively considered since, with proper management, it is related to slightly worse short-term outcomes but long-term results similar to “normal size” grafts [44].

### 3.2. Rejection of the Graft

By the onset time, the rejection is classified into hyperacute (within hours after LT), acute (within two to six weeks), and chronic rejection [26].

The hyperacute rejection rate is very low, and it is the consequence of the presence of specific preformed recipient antibodies leading to low rates of graft survival. ABO incompatibility is a significant risk factor. Clinical manifestations are similar to ischemic graft injuries. An urgent re-transplant is required [27].

Acute rejection is the result of the T-lymphocyte response [27]. It is the most frequent type of graft rejection and affects 15–25% of LT recipients. Patients treated with tacrolimus (Tac), older recipients, and those who had cirrhosis due to alcohol abuse seem to develop this complication more rarely [47]. Symptoms are non-specific and include fever, pain in the right hypochondrium, jaundice, malaise, and alteration in bile production. Laboratory signs comprehend increased cholestatic enzyme levels. A biopsy showing typical findings is considered the gold standard for the diagnosis, and it should be used to confirm the unresponsiveness to the therapy or rejection recurrence. The biopsy can be performed with a percutaneous or transjugular approach. The first-line treatment includes the use of steroids (a bolus of a high dose of methylprednisolone, followed by maintenance with prednisone). Doses of immunosuppressive therapy (IS) are usually increased [27].

Chronic rejection is identified with the so-called ductopenia (loss of bile ducts) and with the obstruction of the arterioles by macrophages. A liver biopsy is required to confirm the diagnosis. Traditional definition required a bile duct loss in more than 50% of the portal triads (at least 20 portal triads must be analyzed) which are present in a late stage of the disease. An extension of the definition allowing an earlier recognition of the problem includes obliterative arteriopathy, atrophy of the biliary epithelium, pyknosis in most of the small ducts, and lower bile duct loss. Differential diagnosis from most of the other causes of cholestasis is usually simple. A cholangiography could help in differentiating chronic rejection from tacrorosing cholangitis (PSC) [48]. More recently, although reported in a small retrospective series, the detection of signs including periportal edema (other than biliary dilatation, ascites, and hepatosplenomegaly) at a CT or MRI scan at least 1 year after LT can suggest the presence of chronic rejection [49].

This complication may be the consequence of recurrent acute rejections, graft ischemia related to artery stenosis, Cytomegalovirus (CMV) infection, or chronic rejection because of antibody-mediated response. Steroids are useless in this condition, and a re-transplantation is often required if graft loss occurs [27]. The use of high doses of tacrolimus in the initial development of this condition has been reported as efficacious [50].

### 3.3. Post-Transplant Infections

Before transplantation, all the recipients should be screened for acute or chronic bacterial, fungal, or viral infections to prevent a potential recrudescence under an IS regimen. LT candidates should be tested, as appropriate, for human immunodeficiency virus (HIV) 1 and 2 (which is no longer an absolute contraindication to LT), hepatitis A, B, and C virus, CMV, *Mycobacterium tuberculosis*, Epstein–Barr virus (EBV), human herpes virus 8 (HHV-8), varicella zoster virus (VZV), herpes simplex virus 1 and 2, Toxoplasma gondii, Treponema pallidum and other venereal disease. In case of aspergillosis infection, an effective treatment is mandatory before LT. Other specific tests should be performed according to the local epidemiology of the recipients, for example, screening for coccidioidomycosis and secondary prophylaxis should be performed in recipients coming from areas in which this infection is endemic. Further specific details could be found elsewhere [51].

Prevention includes vaccination, prophylaxis, and pre-emptive therapy. Recommended vaccinations include those against hepatitis A and B virus, VZV, *Pneumococcus*, *H. influenza*, and tetanus [51].

Surgical stress and the use of IS resulted in higher infection susceptibility and the great majority of LT patients will suffer from some kind of infection. Although proper prophylactic therapies, within the first month, bacterial and fungal infections (mostly nosocomial) may occur in the surgical site, in the abdomen, in the bloodstream, and in the urinary or respiratory tract. The most common bacterial species include *Escherichia coli* and *Pseudomonas.* Higher rates of graft loss can be seen associated with intra-abdominal infections [26]. Selective bowel decontamination with non-absorbable oral antibiotics could be useful to prevent bacterial translocation [52]. All the risk factors for *Clostridium difficile* infection should be reduced [52]. Other infections include donor-derived ones [52].

From one month to six months, infections caused by opportunistic pathogens or reactivation of latent infections (for example, CMV, EBV, HHV, *Listeria*, Toxoplasma) can occur due to the reaching of the steady state of IS and could be a cause of higher morbidity and mortality. CMV is the most common of these pathogens. Despite correct prophylaxis and pre-emptive therapies, clinical manifestations of the infection are viremia, bone marrow suppression, colitis, and hepatitis. Oral antiviral therapy (valganciclovir) can be administered in mild infections while intravenous injection (ganciclovir) is required in severe manifestations [53]. Blood cultures are not always a reliable examen to detect an invasive fungal infection. However, while Candida species infections have been shown to be reduced, *Aspergillus* infection rates are higher. Oral fluconazole prophylaxis against Candida is recommended during the first month. *Aspergillus* infection usually starts in the lung and subsequently extends to the central nervous system which should be confirmed with a lumbar puncture. Use of corticosteroids, AKI, blood transfusion, or liver failure are the major risk factors for *Aspergillus* infection thus requiring prophylaxis with inhaled amphotericin B or micafungin [54]. An adequate prophylaxis with trimethoprim-sulfamethoxazole highly reduces the risk of Pneumocystis pneumonia and toxoplasmosis [52,55]. When latent tuberculosis is suspected during the pre-LT screening, 9-month prophylaxis with isoniazid is recommended. Active tuberculosis infection rates are low (less than 2%) but the treatment could interfere with IS [56].

After 6 months from LT, community-acquired infections are the most common including respiratory, urinary tract, or biliary infections [52].

Daily-life suggestions to reduce the risk of infections include frequent hand washing, the use of gloves while gardening/farming, abolition of smoking habit, avoidance of contact with respiratory ill people, avoiding drinking potentially contaminated water (cryptosporidiosis or giardiasis could be found in public fountains) or eating raw foods, life with healthy pets is possible [52].

### 3.4. Other Medical Postoperative Complications

Pulmonary complications are frequent after surgical procedures. Specific risk factors after LT include long-lasting surgery, substantial intraoperative blood loss, use of blood transfusions and significant fluid infusions, pulmonary aspiration, sepsis, and changes in the hemodynamics after liver reperfusion. Patients are usually extubated as soon as possible when the hemodynamic stability can be confirmed. Pleural effusions at a chest X-ray, often occurring on the right side, are frequent and they usually heal without specific medical treatments. In case of persistence, they may be the cause of atelectasis and, consequently, of pneumonia [27]. Hospital-acquired pneumonia affects 5–38% of the patients after LT [57]. Long-lasting mechanical ventilation is considered a risk factor. Targeted antibiotic therapy and a temporary reduction in IS are required [27]. Adult respiratory distress syndrome (ARDS) may occur even after several weeks from LT. It affects 4.5–16% of the patients and the mortality rate is approximately 80%. Treatment aims to support patient ventilation [58]. Pulmonary edema is a rare complication mostly due to renal failure causing fluid overload [57].

Postreperfusion syndrome (PRS) is a consequence of severe ischemia/reperfusion injury of the liver that causes excessive activation of the inflammatory response. PRS affects up to 55% of the patients after reperfusion and its clinical manifestations include a reduction in systemic vascular resistance with hypotension and lowered cardiac output [59].

Acute kidney injury (AKI) is a frequent complication occurring in up to 78% of patients [60], and PRS is an early predictor of AKI as demonstrated by the association between AKI and peak AST [59,61]. Type of donation (ECD including DCD), donor age, high recipient MELD score and BMI, and massive intraoperative need for transfusion are the principal risk factors for AKI development [14,61]. Kollmann et al. performed a retrospective study, including 681 patients over 5 years. They compared 57 patients after DCD with 446 after DBD and 178 after LDLT. DCD had a higher chance of developing AKI (61% vs. 40%) and chronic kidney disease (CKD). Long-term survival was similar, and only severe AKI (which occurred in a small percentage of the patients) resulted in affecting the survival rate. Interestingly, AKI did not impact the probability of developing CKD, whose primary cause seems probably related to the use of calcineurin inhibitors (CNI) as IS [61]. On the contrary, Kalisvaart et al. reported a 1.8-fold increased possibility of developing a CKD after AKI. Furthermore, they found no relation between CKD and the kind of donors [62]. The sum of donor and recipient warm ischemia time (when exceeding 60 min) seems to be associated with AKI [60]. Recently, intraoperative oxygen delivery management during LT has been reported to be a significant time-dependent risk factor for AKI [63]. An AKI predictive score was developed and included donor and recipient BMI, DCD grafts, FFP requirements, and recipient warm ischemia time [64]. The use of ex situ NMP seems to reduce ischemic/reperfusion injury and the chance to develop AKI [22].

Neurologic complications after LT are quite common and may be a cause of postoperative mortality. Although patients usually develop these problems within the first month, neurological complications may occur up to a year after the LT. These complications are multifactorial and include encephalopathy, seizure, and focal motor deficits [65]. Encephalopathy is the most common neurological complication, and it may be caused by impaired graft function, development of sepsis, uremia, use of CNI or steroids, and occurrence of central pontine myelinolysis, a severe pathology with an incidence ranging between 1% and 3% after LT [66,67]. Seizures are the second most common neurologic complication. CNI and steroids are potential trigger factors since they are associated with neurotoxicity. Alteration in the electrolyte or metabolic balance, opportunistic infections (for example, VZV, HHV-6, Toxoplasma, or fungal infection causing brain abscesses), or bleeding affecting the central nervous system are other potential causes. Up to 7% of liver recipients will develop a critical illness myopathy [68]. Post-LT demyelinating inflammatory polyneuropathy is another rare complication [69]. The management of these complications aims to treat the specific cause, whenever possible [27]. Chronic neurological complications include tremors (the most frequent symptom), headaches, insomnia, hearing loss, and paraesthesia [70]. The neurotoxicity of IS is widely studied. Tacrolimus toxicity seems to be higher than cyclosporine. IS dose reduction is usually sufficient, while severe complications may require the use of beta-blockers, calcium antagonists, or tricyclic antidepressants [67].

## 4. Other Long-Term Complications

### 4.1. Cardiovascular Complications

Cardiovascular complications are a frequent cause of mortality with a functioning graft accounting for approximately 20% of the deaths of the patients surviving at least 3 years from LT [71]. For LT recipients, the global risk of cardiovascular events is higher when compared to the general population, and it is reported to be up to 25% 10 years after LT [72,73]. Hypertension is a well-known risk factor. LT recipients may have many additional risk factors for developing hypertension, including renal damage and the use of steroids and CNI. Consequently, the prevalence of hypertension in LT recipients ranges from 36% to 77% [74]. Lifestyle changes are the first approach, while pharmacologic therapy may include the use of blockers of the calcium channel, angiotensin-converting enzyme inhibitors, beta-blockers, or loop diuretics [75]. The use of tacrolimus is related to a lower chance of developing hypertension when compared to the use of cyclosporine. Similarly, hyperlipidemia, diabetes mellitus, and obesity are other risk factors for cardiovascular disease. The prevalence of these conditions is reported to range between 27 and 66% [74]. Poor physical activity, use of steroids, CNI, and sirolimus (for hyperlipidemia) are additional risk factors in developing these conditions. In particular, steroids and CNI could predispose a state of insulin resistance and alter insulin metabolism. Lifestyle advice, close screening, and reduction/avoidance of steroids and CNI should be warranted in the subset of patients having a high risk of cardiovascular events [27].

### 4.2. Metabolic Syndrome

Metabolic syndrome includes hypertension, insulin-resistant diabetes mellitus, obesity, and dyslipidemia. Metabolic syndrome rates are reported to range between 50 and 60% [76]. Up to 25% of the deaths in the long term are caused by cardiovascular accidents, thus requiring a pharmacological and non-pharmacological treatment of each one of these conditions [77]. Other than for cardiovascular pathologies, the syndrome is a risk factor for the development of post-LT NAFLD/NASH [78]. In these patients, the use of corticosteroids should be avoided whenever possible [79].

### 4.3. Kidney Disease and Hyperuricemia

Within 5 years from LT, up to 80% of the patients will develop chronic kidney disease (CKD) and approximately 10% of them will need dialysis/kidney transplantation within 10 years [80]. Due to the use of marginal donors, these percentages are expected to increase in the next years. The use of CNIs is the principal cause since it is related to an initial reversible condition of renal vasoconstriction but then tubulointerstitial chronic fibrosis and other irreversible changes occur [81]. Other risk factors for CKD are advanced age, post-LT CMV infection, pre-LT kidney function impairment, and the development of metabolic syndrome [62]. There are several studies comparing the effect on renal function of the delayed use of Tac showing conflicting results [82,83]. A reduction of half of the Tac dose associated with MMF can improve renal function without increasing the risk of acute rejection. Further lowering Tac dose, too high percentages of acute rejection will be observed [84,85]. A close renal function periodical check is mandatory [79].

Hyperuricemia has been reported in 14–47% of the patients [86]. Since acid uric has a renal excretion of 60–70%, hyperuricemia is related to renal function impairment, but it could also be a cause of renal function further deterioration [87,88]. Indeed, allopurinol use could help in ameliorating the renal function [86]. Again, CNIs determine a higher tubular urate reabsorption or a reduced uric acid glomerular filtration representing another cause of hyperuricemia [87]. Although reported in a small group, hyperuricemia (6.5 mg/dL) seems to be an independent factor for mortality in patients with impaired renal function [87].

### 4.4. Bone Complications

Bone complications are frequent findings in patients with chronic liver disease. Atraumatic bone fractures affected 20–40% of these patients and up to 65% of LT recipients because of cholestatic disease [89]. Hormonal changes, resulting from liver malfunction, are responsible for rearrangement in bone metabolism together with the use of IS and steroid. Classical treatments for osteoporosis (e.g., calcium, vitamin D, and bisphosphonate) are the most used.

### 4.5. Dermatologic Non-Oncological Complications

These complications, mostly related to IS, comprehend a wide range of disorders including xerosis cutis, sebaceous hyperplasia, hyperpigmentation, steroid-induced acne, vascular lesions, hair and nail abnormalities. Most of these disorders frequently appear within the first month from LT but subsequently improve. Furthermore, herpetic infections, or fungal infections (mostly tinea) of the skin and of the nail are frequently found [90].

### 4.6. Recurrence of Viral Hepatitis, Autoimmune Hepatitis (AIH), Primary Biliary Cirrhosis (PBC), and PSC

LT recipients with active HCV infection will experience HCV recurrence. Immunosuppression causes a faster development of the disease with severe risks for graft and patient survival [91]. When pre-transplant HCV eradication is not feasible, antiviral therapy should be initiated as soon as possible, especially in patients with liver fibrosis greater than F2. However, immediately after LT, the risk of renal failure, infections, and cytopenia limited the use of antiviral drugs; therefore, the treatment should be started after pathological confirmation of liver damage [92]. To identify these patients early, a careful follow-up with liver biopsy, portal hypertension evaluation, and transient elastography measurement is mandatory [79]. Furthermore, the estimation of these parameters one year after LT is reported to be a reliable predictor of graft loss [93].

The recurrence of HBV-related liver disease could be predicted with the pre-LT levels of HBV DNA. However, it has decreased in the last two decades due to the use of specific immunoglobulin and nucleoside analogs [79].

Up to half of the patients will suffer from AIH, PBC, or PSC recurrence; however, this eventuality will not significantly modify patient prognosis [94]. A liver biopsy is usually needed to confirm the recurrence and a cholangiography is helpful in the differential diagnosis. The biliary epithelial cell staining with the antibody against the pyruvate dehydrogenase complex is pathognomonic of PBC [48]. Prophylactic use of ursodeoxycholic acid is not recommended [79].

### 4.7. De Novo Malignancies

De novo malignancies are one of the leading causes of mortality with a functioning graft accounting for approximately 21–25% of all deaths [71]. Usually, in LT recipients, tumors have more aggressive biology [95]. This increased susceptibility has two leading causes: recipient factors and lifetime IS. Recipient factors include advanced age, gender and race, alcohol use, smoking habit, and the indication for LT. For example, patients with PSC have a risk of developing non-skin cancer up to 22% at 10 years [96]. Similarly, patients transplanted because of HCC have a probability of developing HCC recurrence up to 20% with high disease-related mortality rates. HCC recurrence mostly occurs within the first 2 years, but it has been described even 25 years after LT [97]. Life-long IS causes a deficit in the immune surveillance system, promoting the survival and proliferation of malignant cells. Furthermore, IS can reactivate latent oncogenic viruses (e.g., EBV, human papilloma, and HHV 8).

Skin cancers are the most common malignancy in LT recipients. Herrero et al. reported a relative risk for skin cancer 20-fold higher in LT recipients [98], mostly related to the degree of IS [99]. The most frequent tumors are squamous cell cancer, basal cell cancer, and Kaposi’s sarcoma. Although quite rare, Kaposi’s sarcoma occurs in LT recipients with an incidence 500-fold higher than in the general population [100].

Post-transplant lymphoproliferative disorders (PTLDs) indicate a broad spectrum of lymphoproliferative pathologies. They are the second most common de novo malignancies with a global incidence of 5–20%, especially in the pediatric population [100]. The risk is highest within the first 18 months after surgery, while the incidence of PTLD lowers to 6% at 15 years after LT. IS and EBV primary infection is a crucial risk factor for PTLD pathogenesis, mostly in younger recipients [101]. On the other hand, non-EBV-related PTLD incidence is increasing. It generally develops later and affects older patients with a worse prognosis. Probable risk factors are older age, HCV or alcoholic cirrhosis, and use of anti-lymphocyte/thymocyte antibodies. The clinical manifestation of PTLD can range from benign polymorphic conditions to aggressive lymphomas, usually with extra-nodal involvement. The one-year mortality rate after PTLD diagnosis is up to 40%, while 5-year overall survival is around 50% [102]. The appropriate treatment is still debated. A multimodal approach with IS weaning (or switch to an mTOR inhibitor [103]), use of radio-chemotherapy, and surgery is generally used to cure and prevent the recurrence of PTLD [104].

Solid organ de novo cancers can seriously affect LT recipient prognosis. Lung cancer is responsible for approximately 26% of all deaths for de novo malignancies after LT. Smoking is the main risk factor. However, the survival rate is similar to the healthy population [105]. Head and neck cancers generally develop between 31 and 50 months after LT. They are less frequent, but they are still burdened by a high mortality rate with a 5-year survival rate of 35% [106]. Gastrointestinal cancers are more common in recipients suffering from inflammatory bowel disease, associated or not with PSC [96], and they usually occur at a younger age. Other de novo malignancies include bladder and breast cancer [107]. Adequate surveillance is crucial to make an early diagnosis. However, no specific and standardized long-term protocols are available in the literature [101].

## 5. Surgical Complications

### 5.1. Hemorrhage

Postoperative bleeding affects up to 5% of LT recipients, usually occurring within 48 h after LT. Hemorrhage is the leading cause of hypotension immediately after LT. A significant risk factor is delayed graft function. Other reasons comprehend, thrombocytopenia (with multifactorial pathogenesis), hypocalcemia, and dilution. In the presence of hemodynamic stability, conservative management may be advocated. On the contrary, a relaparotomy with a careful inspection of the anastomoses is mandatory. However, in the great majority of the patients, the source of the bleeding will not be found. Rupture of a pseudoaneurysm of the extrahepatic portion of the hepatic artery (see below) may be another cause of massive intraperitoneal bleeding [27].

### 5.2. Vascular Complications

Vascular complications have a global incidence of approximately 7%, but they are the cause of high graft loss and mortality rates, especially when diagnosed late [108]. Early diagnosis is mandatory to prevent further complications. A Doppler ultrasound is usually the first diagnostic test. Angio-CT scan or angiography may be required to confirm the diagnosis [109].

The hepatic artery thrombosis (HAT) is the most frequent arterial complication with a reported incidence ranging between 1.9% and 9%, and it is the leading cause of graft loss [108]. Risk factors include damage to the endothelium, prolonged cold ischemia time, the necessity of blood transfusions, recipient hypercoagulability, transplantation in low-volume centers, and technical mistakes. Pediatric recipients have a higher incidence rate of early-onset HAT when compared to adult recipients (42% vs. 12%, respectively) [110]. A French study compared previously discarded liver transplantation with LT from standard allocation. They reported a significantly higher but acceptable rate of HAT and graft loss with similar overall patient survival for the recipients of the previously discarded liver. They underlined the opportunity to reconsider a graft allocation but with careful donor/recipient matching [111]. Thrombosis may occur with early onset (within the first 30 days after LT) or, less frequently, with late onset (more than 30 days after LT) [27]. In the early onset of HAT, there is often fever, mental status alterations, and hemodynamic instability. Laboratory exams show a sudden increase in liver enzymes and INR. Possible consequences are graft ischemia/necrosis, necrosis of the bile duct, formation of an intrahepatic abscess, and multiorgan failure. Without treatments, 50–70% of patients will experience liver graft failure with a mortality rate of more than 50% [108]. Preoperative thromboelastography seems to be an interesting predictive tool for early HAT occurrence with a sensitivity and specificity of 70% and 73%, respectively [112]. Pareja et al. proposed a routinary protocol of screening with a Doppler ultrasound performed within the first two days after LT and after a further seven days [32]. Recently, thrombolysis or thrombectomy with an endovascular approach has been proposed as a first-line treatment [108]. The success rate reaches approximately 100% [113]. In case of failure, an urgent retransplantation is required [27,108]. Late onset of the HAT is not a cause of graft function impairment, and the patients are usually asymptomatic. However, they can develop further biliary complications (intrahepatic biliary stricture, intrahepatic biloma, biliary ischemia, biliary duct stenosis, and sepsis) since the hepatic artery supplies the biliary tree. The treatment aims to resolve the biliary complications and it may include percutaneous drainage, antibiotic therapy, or bilio-enteric bypass. A retransplantation may be required if there are irreversible biliary tract damages [27].

Hepatic artery stenosis (HAS) has an incidence ranging from 0.8% to 10% [108,114]. Possible causes include technical errors, injuries to the intimal layer, and kinking/twisting of the vessel. The prognostic impact of this complication is variable, while some patients may result asymptomatic, up to 50% of the untreated patients may experience a subsequent HAT [27]. Endovascular angioplasty, with or without stent placement, is a feasible and repeatable treatment, but long-term vascular patency and graft survival rates are still discussed [115]. Saad et al., in a study with 37 patients, reported a 44% patency rate 14.5 months after percutaneous balloon angioplasty [116]. More recently, Rajakannu et al., in their series of 30 patients, showed a 90% success rate for the angioplasty. Only one arterial dissection occurred and required retransplantation. The 1- and 5-year patency rates were 68% and 62.8%, 5-year graft survival was 64.5%, and 5-year overall patient survival was 85.3%. Interestingly, stenting placement did not affect the patency rate [117].

Hepatic artery pseudoaneurysm (HAP) is a rare arterial complication with an incidence of 1–3% [108]. LT recipients develop HAP usually in 2 or 3 weeks after LT [118]. The leading cause of HAP is an infection at the anastomotic site leading to the formation of a pseudoaneurysm in the extrahepatic tract. Fungi in the bloodstream and postoperative pancreatitis are other risk factors. The involvement of the intrahepatic segment of the artery is quite rare, and it is usually the consequence of percutaneous procedures. HAP is a life-threatening complication with a mortality rate of 69% since symptoms are often aspecific, including fever, low level of hemoglobin, and malfunctioning of the graft [118]. Further complications include graft ischemia due to artery thrombosis, massive hemoperitoneum due to the rupture of an extrahepatic pseudoaneurysm, or formation of an arteriobiliary fistula with hemobilia due to the rupture of a pseudoaneurysm of the intrahepatic artery [119]. Arteriography is the gold standard for diagnosis, and embolization is a valuable option in hemodynamically unstable patients as a bridge for urgent retransplantation. Surgical HAP resection and reconstruction, usually with arterial graft, is the treatment of choice, but a retransplantation may be needed [118].

Arterial conduit occlusion is a possible complication when the use of the native artery is not possible. The reported incidence is approximately 8%. Donor age greater than 40 years and previous coronary artery bypass seem to be independent risk factors. On the contrary, the use of aspirin is associated with higher patency rates. The placement site of the conduit appears to be irrelevant for conduit patency [120]. Percutaneous endovascular treatments are effective but with a mean 1-year patency rate of 22% [121].

The principal causes of portal vein complications are technical mistakes, including twisting or kinking of a redundant vessel, and low flow through the portal vein [27].

Portal vein stenosis (PVS) has an incidence of less than 5%. A potential risk factor is the use of a split liver: LDLT and pediatric recipients have a reported incidence of PVS up to 27% [63]. If a Doppler ultrasound is performed, a ratio higher than 4:1 between the anastomotic and pre-anastomotic velocity of the flow is suggestive of vein stenosis with high specificity [119]. A percutaneous transhepatic or transjugular angioplasty may be a valuable alternative to the surgical resection and reconstruction of the portal vein [122].

Portal venous thrombosis (PVT) has an incidence of 2–3%. A state of pre-existing hypercoagulability is an additional risk factor [27]. Clinical manifestations include sudden symptoms and signs of acute liver failure (coagulopathy, hypoglycemia, lactic acidosis), signs of portal hypertension (massive ascites and bleeding from esophageal varices), renal failure, and hemodynamic instability. Surgical vein resection with thrombectomy and direct anastomosis with the use of anticoagulant drugs are the treatment of choice.

Venous outflow complications caused by hepatic vein or vena cava stenosis have an incidence ranging from 1% to 6%, mostly occurring during the first weeks after LT. Possible causes are tight anastomosis, a discrepancy between the size of the vein of the donor/recipient (as in pediatric recipients after LDLT), twisting or compression of the vein, the formation of an intimal vein flap, and kind of anastomotic technique [27,108]. The modified piggyback with the three veins technique is related to lesser vein outflow complication rates [123]. Clinical manifestations are mostly the same as those seen in portal hypertension. Further complications may be renal and liver graft impairment. A pressure gradient of 10 mmHg at a Doppler ultrasound evaluation is considered a cut-off for the diagnosis of venous outflow stenosis [124]. Percutaneous transjugular balloon angioplasty with the placement of a stent is the preferable treatment for these complications. The reported technical and clinical success rate ranges from 78% and 100%; stent positioning allows for higher patency rates, but the risk of anastomotic rupture is higher [125]. Surgical revision of the anastomosis may be necessary if the vessel stenosis persists.

### 5.3. Biliary Complications

Biliary complications are quite common after LT, with a global incidence ranging from 2% to 19% [126]. These complications are related to significant postoperative morbidity and mortality rates after LT [127,128]. The most common biliary complications include biliary leakage, and biliary strictures [128]. Less frequent complications are intrahepatic strictures, papillary dysfunction, biliary strictures caused by stones, and cystic duct mucoceles [128,129]. The risk factors for these complications include characteristics of the donor (i.e., the small caliber of the ducts, donor age, and infectious disease), surgical technique, and procedures of graft preservation [128]. A Doppler ultrasound evaluation should always be performed to diagnose these biliary complications and to rule out arterial inflow issues.

Bile leaks (BL) have an incidence of 8.2% [128]. They usually occur within the first month after LT, although some authors reported the occurrence of BL up to 6 months after surgery [126]. Possible sources of leakage are the biliary anastomosis, T-tube insertion site (if used), the stump of the cystic duct, damage on the liver surface, or liver section surface (split liver transplantation). Technical mistakes, including the tension between the stumps or necrosis of the bile duct because of HAT, are specific risk factors for BL at the anastomotic site [27]. The choledocholedocostomy (CC) is burdened by lower BL rates than hepaticojejunostomy, and if a BL occurs, there will be no enteral contamination of the abdomen. Furthermore, cholangitis occurrence is lower after CC [26]. Clinical manifestations may vary from the absence of symptoms to fever and abdominal pain. Conservative treatment can be chosen in asymptomatic patients who may benefit from biliary decompression through T-tube opening. Furthermore, radiology-guided positioning of abdominal drains may be useful [126,127].

Extrahepatic biliary strictures are the most common biliary complications affecting up to one-third of the patients, mostly within the first year after LT [128]. The presence of edema, twisting of the anastomotic stump, BL, arterial complications (including HAT or HAS), and biliary infection represent specific risk factors for extrahepatic biliary strictures. A recent study analyzed the role of the peribiliary gland (PBG) in causing extrahepatic nonanastomotic strictures. PBG harbors stem cells involved in biliary regeneration. PBG and peribiliary vascular plexus may be damaged during LT, determining an excessive activation of cell proliferation and VEGF-A expression and causing biliary stenosis [130]. Laboratory exams show an increase in the level of cholestatic enzymes. CT scan and magnetic resonance cholangiopancreatography are valuable diagnostic tools. For the recipients who received a duct-to-duct anastomosis, the endoscopic treatment (through an endoscopic retrograde cholangiopancreatography—ERCP) with the sphincterotomy and the positioning of a trans-papillary biliary stent represents the standard of care for both strictures and leaks, and it should be preferred over the percutaneous and surgical approaches [127]. A nasobiliary drainage to decompress the biliary system may be another additional useful treatment [126]. The percutaneous approach is mandatory if a Roux-en-y choledochojejunostomy has been performed. This treatment may be challenging if the biliary tree is not dilated. Endoscopic and radiologic techniques can be repeated over time, they are generally safe and often useful, especially for short-term outcomes, and they can be used as a bridge to a definite surgical treatment. On the other hand, the surgical reconstruction of the anastomosis is necessary if the other treatments fail or if peritonitis occurs [128]. A surgical approach for nonanastomotic strictures with a biliary reconstruction may improve graft function and patient condition mostly when stenosis occurs within the first 2 years from LT, suggesting that after 2 years from LT, a reduction in bile outflow is not the cause of graft dysfunction [131].

Intrahepatic biliary strictures, also known as ischemic-type biliary strictures or ischemic cholangiopathy (IC), are worrisome biliary complications with an incidence ranging from 3% to 16%. IC usually occurs within the first 6 months [129,132]. Approximately 65% of patients will require a retransplantation [133]. They are a consequence of ischemic injuries, mainly affecting the biliary epithelium and the peribiliary arterioles, which leads to the development of fibrosis. Other causes include immune-related processes (ABO incompatibility or chronic ductopenia), opportunistic infections (i.e., cytomegalovirus), recurrent hepatitis (B or C), primary sclerosing cholangitis (PSC), bile salt toxicity, nodular regenerative hyperplasia, and post-transplant lymphoproliferative disorder or other tumors. Recipients from marginal donors with prolonged warm and cold ischemia time, extended (more than 12 h) preservation of the graft, and HAT sequelae are specific risk factors for developing IC [129]. IC rates were generally reported to be higher in DCD when compared to DBD (10–16% vs. 3%) but without the impairment of graft function as confirmed by similar graft survival rates [14,133]. Several studies are reporting comparable IC rates after DBD or marginal donor grafts treated with NRP and/or grafts recovered with ex situ MP. The benefit of the use of NRP is still debated [17,133]. The use of ex situ NMP maintains physiological pressure and flow rates within the graft, reducing hypoxia and ischemic injuries. Consequently, NMP seems to allow a reduction in the biliary complication rate, up to 2.5% [20,133]. The use of HOPE ensures nourishment and oxygenation of the liver, and eliminates cytokines and toxins from the liver, thus decreasing graft injuries. Dutkowski et al. performed a retrospective comparison matching the results of grafts recovered with SCS or HOPE. IC rates were 22% and 0%, respectively, and 1-year graft survival rate was 69% and 90%, respectively. Similarly, lower rates of PNF and other biliary complications were found in the HOPE group [24]. Some other authors reported the superiority of HOPE vs. NMP in terms of IC and other short-term complications [22]. Hypothermic perfusion allows for a reversible suppression of mitochondrial metabolism, reducing the production of reactive oxygen species, the activation of the inflammatory response, and the postreperfusion syndrome occurrence [24]. All these studies may suffer from selection bias, and further investigations are needed.

Since there is an enormous heterogeneity of behavior regarding bile duct and vascular perfusion (quality, quantity, and modality of the use of the perfusate), and the use of other drugs in the donor (including fibrinolytic agents, heparin, steroids, or prostacyclin), definite conclusions about the potential risk factors related to organ procurement and transplantation cannot be drawn [132]. However, the harmful effect of the residual bile during cold graft preservation has been demonstrated, biliary duct rinse should always be performed, and further contamination of the bile should be avoided [132]. On the other hand, there are no data demonstrating the superiority of a preservation solution over another or about the exact volume needed [134,135]. Heparin administration seems promising especially before circulatory arrest in DCD (where legally permitted), allowing the reduction in thrombus formation and adequate perfusion of the peribiliary capillary plexus [132]. Again, further studies are needed to confirm these data, and there is an ongoing RCT analyzing the impact of the use of thrombolytic agents based on previously published data [136].

Clinical presentation of IC includes malaise, mild fever, cholestasis, or even septic shock [133]. The diagnosis is made with cholangiographic imaging. A percutaneous approach could be attempted, but the related failure rate is high with a prolonged hospital stay, increased costs, and a great chance of graft loss. Consequently, retransplantation is usually required [127].

## 6. Quality of Life

Lastly, LT recipients experience a decrease in their quality of life (QoL). Evaluation of QoL has been neglected for a long time, and no standardized studies are available. The literature reports that perception of a new life and new perspectives overcome depression and anxiety within the first year after LT. Still, the side effects of IS worsen the initial satisfaction from the second year [137]. However, the 10- and 30-year perception of QoL seems quite good [138]. The great majority of LT recipients suffer from muscle loss because of cirrhosis. The recovery (not always complete) from this condition of frailty may occur up to 1 year after LT. However, physical activity after LT should be encouraged to reduce the risk of cardiovascular events, the onset of post-transplant metabolic syndrome, and to improve short- and long-term QoL impacting both physical and mental status [137].

Female recipients in reproductive age need adequate counseling about the possibility of pregnancy. EASL guideline recommends that pregnancy should be avoided for the first 12–24 months after LT. After this period, pregnancy is possible without the need for IS withdrawal [79].

Finally, although the use of IS is related to the majority of long-term post-LT complications impairing QoL and some clinical trials explored this aspect, its total progressive withdrawal is still experimental and it could not be recommended [79].
